# Validation and forensic application of a new 36 X-chromosomal short tandem repeat loci multiplex system

**DOI:** 10.1093/fsr/owae029

**Published:** 2024-04-23

**Authors:** Kaiqin Chen, Ruiyang Tao, Yiling Qu, Junnan Lu, Yuan Ping, Yilun Zhang, Pengyu Chen, Chengtao Li

**Affiliations:** Key Laboratory of Cell Engineering of Guizhou Province, Clinical Stem Cell Research Institute, Affiliated Hospital of Zunyi Medical University, Zunyi, China; Shanghai Key Laboratory of Forensic Medicine, Shanghai Forensic Service Platform, Academy of Forensic Sciences, Key Laboratory of Forensic Science, Ministry of Justice, Shanghai, China; Shanghai Key Laboratory of Forensic Medicine, Shanghai Forensic Service Platform, Academy of Forensic Sciences, Key Laboratory of Forensic Science, Ministry of Justice, Shanghai, China; Shanghai Key Laboratory of Forensic Medicine, Shanghai Forensic Service Platform, Academy of Forensic Sciences, Key Laboratory of Forensic Science, Ministry of Justice, Shanghai, China; West China School of Basic Medical Sciences and Forensic Medicine, Institute of Forensic Medicine, Sichuan University, Chengdu, China; Public Security Bureau of Wujin District of Changzhou, Changzhou, China; Ningbo Health Gene Technologies Co., Ltd, Ningbo, China; Ningbo Health Gene Technologies Co., Ltd, Ningbo, China; Key Laboratory of Cell Engineering of Guizhou Province, Clinical Stem Cell Research Institute, Affiliated Hospital of Zunyi Medical University, Zunyi, China; Shanghai Key Laboratory of Forensic Medicine, Shanghai Forensic Service Platform, Academy of Forensic Sciences, Key Laboratory of Forensic Science, Ministry of Justice, Shanghai, China; Institute of Forensic Science, Fudan University, Shanghai, China

**Keywords:** forensic genetics, X-chromosomal short tandem repeat, validation study, Chinese Han

## Abstract

X-chromosomal short tandem repeats are indispensable in specific cases, distinct from autosomal and Y chromosome genetic markers. SureID^®^ X37 is an innovative six-colour fluorescence multiplex detection system that can simultaneously amplify 36 X-chromosomal short tandem repeat loci (including DXS6795, DXS7132, DXS8378, DXS10101, DXS10103, DXS10079, DXS10134, GATA165B12, GATA172D05, HPRTB, DXS6810, DXS10135, DXS6797, DXS10074, DXS7424, DXS9902, DXS7423, DXS10148, DXS10162, DXS6809, DXS10159, GATA31E08, DXS6803, DXS10075, DXS6807, DXS10164, DXS6789, DXS10146, DXS7133, DXS6804, DXS981, DXS9895, DXS101, DXS6800, DXS9907, and DXS8377) and the Amelogenin locus. In this study, we validated its suitability for forensic identification per Scientific Working Group on DNA Analysis Methods guidelines, including PCR conditions, precision, accuracy, case-type samples, sensitivity, repeatability, reproducibility, species specificity, stability, stutter calculation, and DNA mixtures. Additionally, 577 Chinese Han individuals were used to investigate the utility of the system in forensic population genetics. The results indicated that the system is sensitive, stable, and reliable and is suitable for typical forensic cases. Subsequent population investigations confirmed that it serves as a potent supplementary tool in forensic applications.

## Introduction

Short tandem repeat (STR) sequences are DNA fragments with polymorphic fragment lengths, characterized by their widespread distribution, high sensitivity, and significant genetic variability. With time, STRs have emerged as the most commonly used genetic markers in forensic science, and since the initial reports on HPRTB and HUMARA, researchers have continually identified a growing number of X-STR loci [1]. Additionally, advancements in sequencing technology and the emergence of specific cases for its use have attracted increased attention from geneticists towards the X-STR genetic marker [2–4]. Given its distinct inheritance pattern (sex-linked inheritance) and the unique distribution of allele frequencies among populations, the X-chromosome marker serves as a valuable complement and enhancement to identification methodologies based on autosomal and Y chromosome markers [5–7]. The unparalleled role of X chromosome genetic markers has been evident in various scenarios, including cases of gang rape, specialized cases, complex kinship identification (i.e., incestuous relationships, single-parent missing cases, and the identification of unique materials), as well as in the domain of population genetics research [8–16].

However, as the research progressed, certain limitations of X-STR markers have become increasingly apparent [3, 4]. The length of the X chromosome is 155 million base pairs (~180 cm), and to date, only 85 STR loci have been identified on the X chromosome, with most of them lacking in-depth investigations [4]. Furthermore, the application of X-STR loci exhibits regional specificity, and there are diverse existing typing methods. Moreover, compared to other countries, China has conducted limited research on X-STR loci, and the available population genetic data are also limited. Thus, it is important to develop a multiplex PCR system tailored to the Chinese population and expand the inclusion of highly polymorphic X-STR loci within this composite system to address the evolving needs of forensic cases and the demand for constructing relevant databases [17].

The six-colour fluorescence detection kit, SureID^®^ X37, has been developed to include most X-STRs in mainstream X chromosome kits available in the market, enabling the simultaneous amplification of 36 X-STR loci (including DXS6795, DXS7132, DXS8378, DXS10101, DXS10103, DXS10079, DXS10134, GATA165B12, GATA172D05, HPRTB, DXS6810, DXS10135, DXS6797, DXS10074, DXS7424, DXS9902, DXS7423, DXS10148, DXS10162, DXS6809, DXS10159, GATA31E08, DXS6803, DXS10075, DXS6807, DXS10164, DXS6789, DXS10146, DXS7133, DXS6804, DXS981, DXS9895, DXS101, DXS6800, DXS9907, and DXS8377) in a single PCR reaction, including the sex-determining locus, Amelogenin. The entire PCR reaction can be completed within 90 min, and the amplified fragments are reasonably sized, typically within 500 base pairs.

Before the implementation of SureID^®^ X37 in forensic applications, comprehensive validation experiments were imperative. In this study, we conducted a series of validation studies on the SureID^®^ X37 detection kit to assess its reliability and suitability for forensic applications following the “DNA Validation Guidelines for DNA Analysis Method” (2016) issued by the Scientific Working Group on DNA Analysis Method (SWGDAM) [18] and the Chinese National Standard GB/T 37226-2018 (Criterion of Forensic Science Human Fluorescence STR Multiplex Amplification Reagent) [19]. These validations included PCR conditions (annealing temperature, amplification cycle number, and final extension time), accuracy, sensitivity, species specificity, typical sample adaptability, repeatability and reproducibility, consistency, mixture analysis, stability, stutter percentage, heterozygote peak height ratio (PHR), and population investigation.

## Materials and methods

### DNA samples

DNA samples of 9948 and 9947A (Suzhou Xin Hai Biotechnology Co., Ltd, Suzhou, China) were used for sensitivity, mixed, and PCR programme research.

Ten typical biological samples (saliva, oral swabs, peripheral blood/spots, hair follicles, vaginal secretions, menstrual blood, semen, muscle tissue, and costal cartilage) were used for case-type sample research. DNA from several common animal species, including 10 non-primates (chicken, duck, goose, pig, cow, horse, sheep, dog, rat, and rabbit), and one non-human primate (macaque) which animal were used for species-specific research.

A total of 577 peripheral blood samples (316 males and 261 females) were collected from health-unrelated individuals in the Chinese Han population. Genomic DNA was extracted using the QIAamp DNA Blood Mini Kit (Qiagen, Hilden, Germany) and quantified using the NanoDrop 2000 spectrophotometer (Thermo Fisher Technologies, Wilmington, DE, USA) according to the manufacturer’s recommendations. If necessary, the template DNA was diluted to 1 ng/μL using Tris-EDTA (TE) buffer.

### PCR amplification and CE detection

The PCR system comprised 5 μL of SureID^®^ X37 master mix, 2.5 μL of SureID^®^ X37 primer mix, and 1 μL of template DNA, with nuclease-free water added to reach a total reaction volume of 10 μL. For direct amplification samples, a 1.2-mm punch disc was employed instead of template DNA. Amplification was conducted using a GeneAmp 9700 PCR system (Thermo Fisher) equipped with a gold-plated silver 96-well block in “Max Mode”. The amplification cycle parameters were set as follows: an initial denaturation at 95°C for 5 min; 2 cycles at 94°C for 10 s and 63°C for 90 s; 26–28 cycles at 94°C for 10 s and 61°C for 90 s; a final extension at 60°C for 15 min; and then incubation at 4°C.

For the initial electrophoresis, we used the HGT 6-Dye Matrix Standard (plus) dye set (HaierShi Gene Technology Co., Ltd, Ningbo, China) to perform spectral calibration for each fluorescent dye, following the provided instructions. ILS-500 served as the internal size standard, featuring fragments of lengths 70, 87, 100, 125, 150, 175, 200, 225, 250, 275, 300, 325, 350, 375, 400, 425, 450, 475, and 500. Electrophoresis samples were prepared by combining 1 μL of PCR product or allelic ladder with a mixture of 9 μL of 1:40 internal standard and deionized formamide (Thermo Fisher). Subsequently, samples were separated and detected using POP4 polymer (Thermo Fisher) and 36 cm capillaries on an ABI 3500xL genetic analyzer (Thermo Fisher). The electrophoresis conditions were as follows: 24 s for injection at 1.2 kV and 1500 s for electrophoresis at 1.5 kV. Electrophoretic data were collected using GeneMapper ID-X software v1.5 (Thermo Fisher) and compared to the allelic ladder to determine the genotype. Unless otherwise specified, the minimum analysis threshold for allelic calling in the analysis software was set at 100 relative fluorescence units (RFUs).

### Verification of performance

#### Evaluation of PCR conditions

To optimize the PCR conditions for the composite system, a systematic evaluation was conducted. Initially, 1 ng of control DNA 9948 was amplified with different annealing temperatures (59°C, 60°C, 61°C, 62°C, and 63°C) under the recommended 26 cycles. Subsequently, five gradient cycle variations (24, 26, 27, 28, and 30) were tested at the established optimized annealing temperature. Finally, various extension times (0, 15, 30, and 45 min) were also tested to ensure adequate time for Taq polymerase to adenylate the fragments. Each of these experiments was conducted in triplicate, systematically varying a single parameter at a time.

#### Accuracy study and sizing precision

We amplified and genotyped DNA samples from 20 individuals using the optimized PCR conditions to assess accuracy and calculated the differences in size between each allele and its corresponding allele in the allelic ladder based on the genotyping data. Size precision was evaluated by computing the mean base pair sizes and standard deviation (SD) of each allele from 24 injections of the allelic ladder on the same 3500xL genetic analyzer.

#### Sensitivity

Control DNA 9948 was subjected to a series of dilutions and three repeated PCR for sensitivity assessments. The template amounts of the input system were 1, 0.5, 0.25, 0.125, 0.0625, and 0.03125 ng, respectively. We set the analysis threshold of the GeneMapper ID-X software to 50 RFU and used the minimum template quantity with no allele dropout in the genotypes as the detection sensitivity of the reagent kit. Finally, we calculated the average number of alleles and peak heights detected at each reaction volume.

#### Case-type samples, repeatability, and reproducibility tests

The system underwent two rounds of testing on 10 biological samples to assess its suitability and consistency for case-type samples. Another person in an independent laboratory used the same reagent kit to examine the reproducibility of results on the same batch of samples. Additionally, the system’s effectiveness in detecting degraded DNA was evaluated using old bloodstains preserved on (Fluorescence Treated Alumina) FTA cards for periods exceeding 1 and 6 years and paper tissues stored for over 13 years.

#### Concordance evaluation

For the consistency assessment, we initially tested four distinct samples (saliva, hair, menstrual blood, and peripheral blood) from the same individual using the kit to assess the consistency of genotyping among various tissue types. Then, five samples were randomly selected from the case-type samples, along with DNA 9948 and 9947A, as test samples to perform genotyping using the Goldeneye^™^ DNA identification system 21X (Goldeneye Technology Co., Ltd., Beijing, China). We compared the genotyping results with those of the same samples from the case-type samples section to establish their consistency.

#### Mixture study

Nine mixed samples with known ratios (1:1, 1:2, 2:1, 1:4, 4:1, 1:8, 8:1, 1:19, 19:1) were prepared using DNA samples 9948 and 9947A, each with a total amount of 1 ng. Subsequently, we conducted triplicate tests on 1 μL of each mixed solution using the reagent kit. Genotyping results were obtained using 50 RFU analysis threshold. We calculated the proportion of unique alleles of the minor components recovered at each ratio.

#### Species specificity

To investigate species specificity, DNA from several common animal species was subjected to amplification using the new system, with each PCR reaction containing 1 ng of DNA template.

#### Stability study

For the stability assessment, the 36 X-STR kit was used to co-amplify 1 ng of DNA 9948 and various common PCR inhibitors (heme, melanin, humic acid, indigotin, and carbamide) typically encountered in forensic practice. The concentrations of inhibitors were as follows: heme (10, 18, 22, 24 ng/μL); melanin (15, 30, 40, 50 ng/μL); humic acid (12, 20, 28, 32 ng/μL); indigotin (2.5, 5, 7.5, 10 ng/μL); carbamide (150, 300, 450, 600 ng/μL). Each reaction was conducted in triplicate. The tolerance of the kit to inhibitors was evaluated by calculating the ratio of detected alleles and the average peak height for each reaction.

#### Heterozygote balance study

To evaluate the heterozygote balance of the system, we utilized a genotyping dataset of 103 randomly selected females and calculated the value by dividing the fluorescence signal value of the lower allele peak by the higher allele peak at each heterozygous locus.

#### Stutter analysis

Using genotyping datasets from 104 samples, we assessed the expected stutter ratios for each locus within the system by calculating the ratio of stutter peak height to the true allele peak height at each locus. To ensure the capture of all stutter peaks, the analysis threshold for stutter peak height was set to 50 RFU. Additionally, a stutter filter (average stutter ratio plus 3 × SD) was implemented to adjust the parameter settings in the GeneMapper ID-X software.

#### Population investigation and statistical analysis

Amplification was conducted on a cohort of 577 random individuals described in the “DNA Sample” section to investigate the genetic polymorphism and forensic efficiency of the 36 X-STR loci. Arlequin v3.5 software [20] was used to calculate linkage disequilibrium (LD) in both males and females and assess Hardy–Weinberg equilibrium (HWE) exclusively in females. Haplotype frequencies were directly derived from population data. Allele frequencies in males, females, and combined populations were calculated using Modified-PowerState software (Promega, Madison, WI, USA) [21]. Chi-square tests were conducted in the SPSS software version 27.0 (IBM Corp., Armonk, NY, USA) to evaluate differences in allele frequencies between males and females. Furthermore, the online tools available on the ChrX-STR.org 2.0 database (https://www.chrx-str.org/xdb/calculate.jsf) were used to compute polymorphic information content (PIC), power of discrimination (PD) in both females and males, as well as paternity exclusion chance for duos and trios.

## Results and discussion

**Figure 1:**
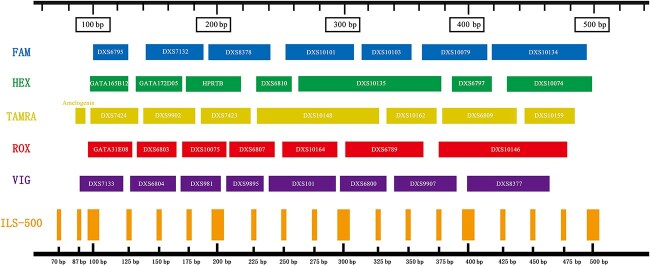
The distribution of loci in the panel.

The detailed information, including locus name, physical distance, repeat motif, etc., and the genotypes of DNA 9948 and 9947A, are shown in Supplementary Table S1. Figure 1 illustrates the distribution of different fluorescent marker sites in the panel based on fragment size.

As shown in Supplementary Figure S1, the profiles of the allelic ladder and internal standard are clear and complete, consistent with the results stated in the manual. In addition, the genotype of control DNA 9948 is consistent with the manufacturer’s instructions (Supplementary Figure S2).

### Evaluation of PCR conditions

The optimal annealing temperature can ensure the targeted PCR product. Overall, we achieved complete profiles at all tested annealing temperatures (Supplementary Figure S3), with the average peak heights ranging from 706.68 RFU at 63°C to 1 845.81 RFU at 59°C. However, allele peak heights were lower at 60°C and 63°C (ranging from 200 to 500 RFU), and the fluorescence balance was suboptimal at 59°C and 62°C (17.49% and 22.48%, respectively). The most favourable combination of allele peak heights (~1 000 RFU) and balance was observed at 61°C. Consequently, we determined that 61°C was the optimal annealing temperature, consistent with the manufacturer’s recommendations. Similarly, all alleles were successfully obtained at all tested cycle numbers (Supplementary Figure S4). At 24 cycles, overall allele peak heights were low, and at 30 cycles, the fluorescence balance was inadequate (~8%). However, suitable allele peak heights and balance were consistently observed at 26–28 cycles. Existing literature has shown that a poor fluorescence balance can increase result uncertainty, as alleles with low fluorescence signals may be considered noise or artefacts, while high signals can introduce non-target peaks that interfere with evidence interpretation [22, 23]. To ensure reliable typing results, we recommend selecting a cycle number between 26 and 28 based on the amplification volume and input DNA amount. During the final extension step, Taq polymerase tends to add a single nucleotide at the 3′ end of the sequence, resulting in PCR products that are one base pair longer than the actual target sequence [24, 25]. Inadequate extension time can lead to irregular peaks in the electropherogram, such as the “-A peak”, complicating genotype assignment [26]. Our experiments with different extension times revealed that at 0 min, several loci (DXS6795, Amelogenin, DXS7132, DXS7424, and DXS9907) exhibited distinct “-A” peaks (Supplementary Figure S5). However, no “-A” peak was observed in the target allele at 15 min, and this pattern remained constant with increasing extension time. Therefore, we recommend using an extension time of 15 min or longer, depending on specific conditions, to ensure accurate genotyping results.

### Accuracy study and sizing precision

**Figure 2:**
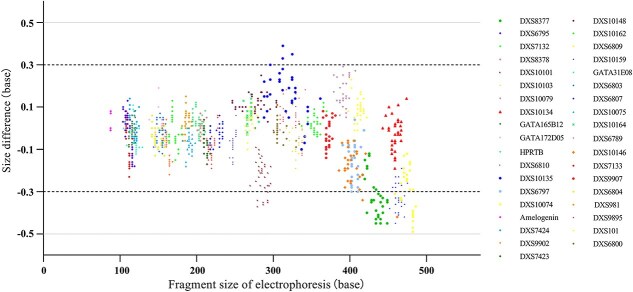
Accuracy study of the SureID^®^ X37 kit. The black dashed and grey solid lines parallel to the *X*-axis represent the differences in size of ±0.3 bp and ±0.5 bp, respectively.

The accuracy test results revealed that the size difference between the alleles of all samples and the corresponding alleles of the allelic ladder was within the range of ±0.5 bp (within the tolerance range of the detection platform), and most of them were even within ±0.3 bp (Figure 2).

**Figure 3:**
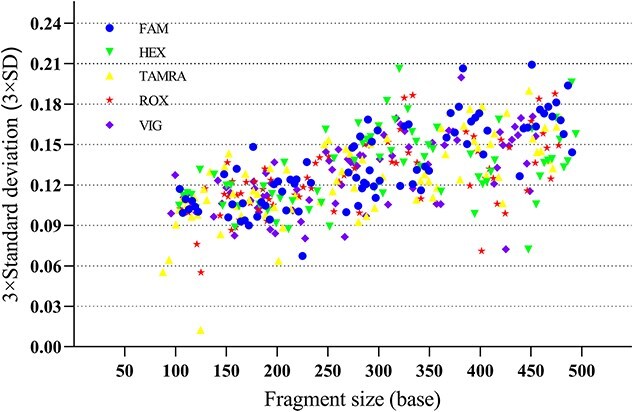
Size precision results of the SureID^®^ X37 kit.

Given that STR genotyping relies on the measurement of fragment size of the allelic ladder concurrently with each sample, assessing size precision is important. As shown in Figure 3, the maximum SD value observed was 0.070 for allele 34 of DXS10134. Furthermore, the fragment size of allele 17 (124.72–124.73 bp) at the DXS7424 locus was almost consistent across all 24 allelic ladder samples, with an SD value of 0.004. These results indicate the high precision of the detection system in enabling accurate discrimination of minor variations, such as partial repeat fragments, within alleles.

### Sensitivity

**Figure 4:**
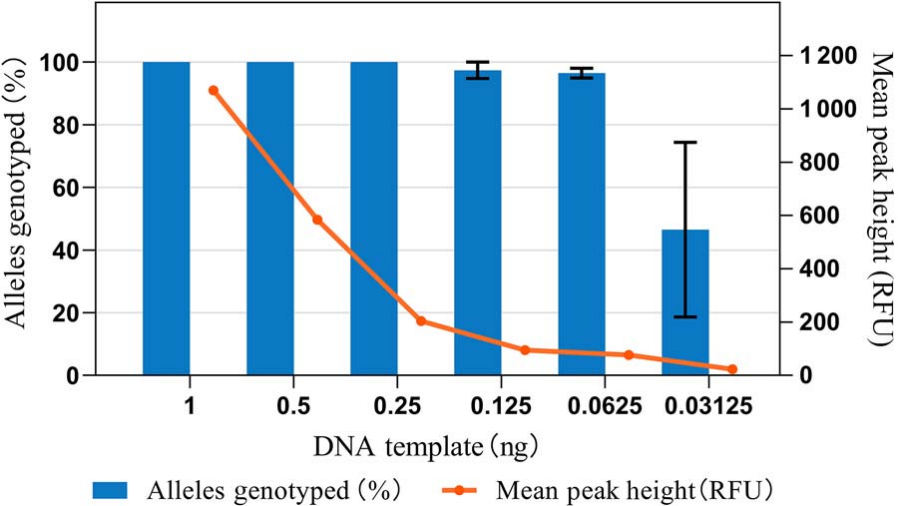
Sensitivity assessments of the SureID^®^ X37 kit. Sensitivity assessments were conducted by template DNA 9948 (1–0.03125 ng). Error bars represent the standard deviation of three repeated experiments.

Forensic experts often need to handle trace DNA samples in practice. To assess system sensitivity and determine the optimal DNA input range for forensic applications, we conducted experiments using diluted DNA 9948, with each sample undergoing three genotyping replicates. Under a peak detection limit of 50 RFU, we observed decreasing average allele peak heights as the input amount decreased (Figure 4). For input DNA quantities between 1 and 0.25 ng, all allele peaks remained detectable, with average peak heights ranging from 1070.12 to 203.73 RFU. At a 0.125 ng template amount, we observed “X” allele dropout at the Amelogenin locus. Further reductions in input amount resulted in average allele peak heights of 75.91 RFU (at 0.0625 ng) and 22.30 RFU (at 0.03125 ng), accompanied by corresponding observed allele frequencies of 96.49% and 46.49%, respectively. Prior studies [27] reported increased randomness in both the PCR reaction and electrophoresis as DNA input decreased, consistent with our results in this study. Therefore, in practical forensic scenarios, we recommend using a minimum of 0.25 ng of DNA input to ensure reliable typing results.

### “Case-type” samples, repeatability, and reproducibility

In practical forensic work, various biological samples, including blood, hair, and different kinds of tissues, are frequently encountered. Test results demonstrated that complete genotypes were consistently obtained from nine out of 10 case-type samples, except for saliva samples, and the results were consistent in repeated experiments. Additionally, these results were consistent with those obtained by another operator. Under the allele calling threshold of 50 RFU, a complete profile was obtained even for the low-concentration saliva sample (0.132 ng/μL), highlighting the reliability of the system for repeatability and reproducibility.

Subsequent experiments demonstrated the system’s ability to successfully detect aged bloodstains stored on FTA cards for up to 6 years. However, for aged bloodstains preserved on tissue paper for as long as 13 years, a significant portion of the larger fragments in the profiles were dropped out (Supplementary Figure S6). In summary, our composite system showed reliability and extensive utility in forensic science. Nevertheless, caution is advised when dealing with aged samples subjected to special preservation conditions.

### Concordance study

Consistency among different tissue types was confirmed by observing that the 36 X-STR genotypes derived from saliva, hair, menstrual blood, and peripheral blood samples from the same individual were identical.

Goldeneye^™^ DNA identification system 21X and the SureID^™^ X37 kit shared 21 identical loci (GATA165B12, DXS101, DXS7424, DXS6807, DXS6809, Amelogenin, DXS6803, GATA172D05, DXS6800, DXS10134, DXS7133, GATA31E08, DXS10159, DXS6789, DXS6810, DXS7423, DXS6795, DXS9902, DXS8378, HPRTB, and DXS7132). When DNA 9948, 9947A, and five case-type samples were tested using both kits, complete profiles were obtained for each sample. The genotypes at the overlapping loci between the two kits were entirely consistent within the same sample. These results provide strong evidence of the reliability and accuracy of the SureID^®^ X37 system.

### DNA mixture test

**Figure 5:**
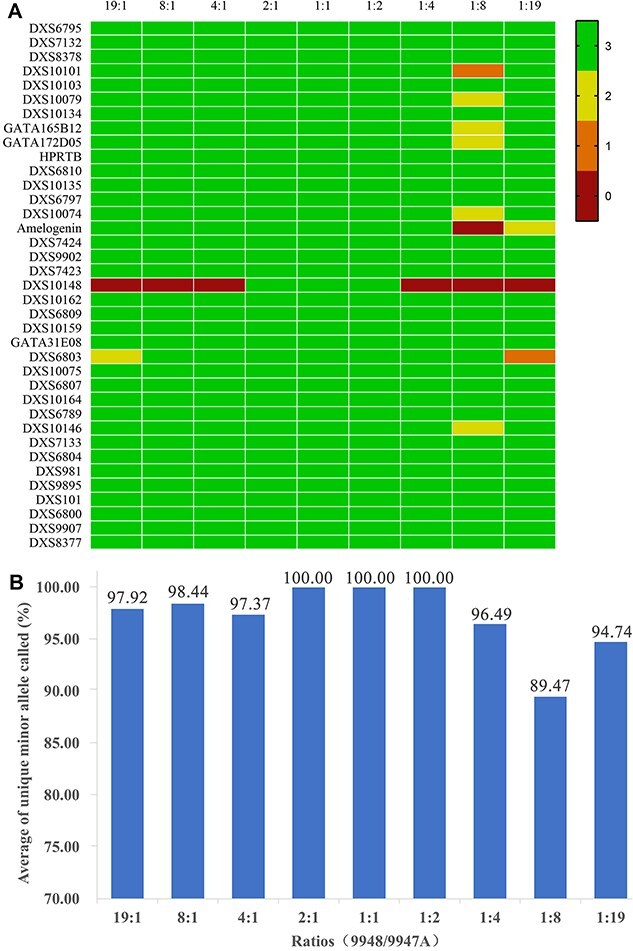
Different proportions of DNA 9948 and 9947A mixtures were studied. (A) Heatmap: Triplicate amplification and detection of each proportion, the number of alleles observed at the locus. (B) Bart chart: An average of unique minor allele called (%) at different ratios of 9948 and 9947A.

In forensic analysis, biological samples often comprise mixtures from multiple individuals, making the interpretation of mixture profiles significantly more complex compared to single-source DNA profiles due to the potential presence of numerous alleles and various intricate features in the profile, including allele dropout and heterozygote imbalance [2, 28–38]. Consequently, it is important to assess the multi-system’s capability to detect mixtures, particularly the profiles of minor contributors. To determine the limit of detection for minor contributors, we analysed non-overlapping alleles in mixtures. The electropherogram of a 1:1 mixture of DNA 9948 and 9947A is shown in Supplementary Figure S7, containing 86 non-overlapping alleles. Minor alleles were detectable at both 2:1 and 1:2 ratios (Figure 5A). However, at 4:1 and 1:4 ratios (Supplementary Figure S7), allele 23 (with the minor component being 9948) and allele 23.1 (with the minor component being 9947A) were not detected at DXS10148. Since 23 and 23.1 were unique to DNA 9948 and 9947A, respectively, they had similar fragment sizes (1 bp). The dominance effect in the same PCR amplification reaction and electrophoresis led to the failure to detect the minor contributor’s alleles. As the mixture ratio increased, the percentage of detectable minor alleles decreased. At ratios of 8:1, 1:8, 19:1, and 1:19, 98.44%, 89.47%, 97.92%, and 94.74% of minor alleles were detected, respectively (Figure 5B). However, when a peak appears at the same position as a stutter peak, it is difficult to determine whether it is a minor component allele peak or a stutter peak. Additionally, as the quantity of minor components decreased, both the stutter peak and heterozygote balance deviated significantly from the norm [22]. Consequently, determining the number of contributors and their corresponding genotypes became more challenging. Therefore, in practical applications, it is essential to use appropriate DNA quantities to ensure accurate genotyping of the samples involved. These findings underscore the system’s potential for effectively analysing mixture profiles.

### Species-specificity studies

Species-specificity studies were conducted using the 36 X-STR system on 11 common non-human DNA samples to assess potential cross-reactivity. None of the non-primate animal profiles displayed any allele peaks. However, in the macaque sample (Supplementary Figure S8), several “OL” alleles with heights ranging from 562 to 1 587 RFU and one “X” allele with a peak height of 217 RFU were observed. This weak cross-reactivity could be attributed to the high degree of genetic homology and close evolutionary relationship between primates and humans, a phenomenon also observed in other studies [39–41]. Overall, the kit demonstrates high specificity for human DNA, but caution is advisable when analysing samples involving primate species.

### Stability studies

**Figure 6:**
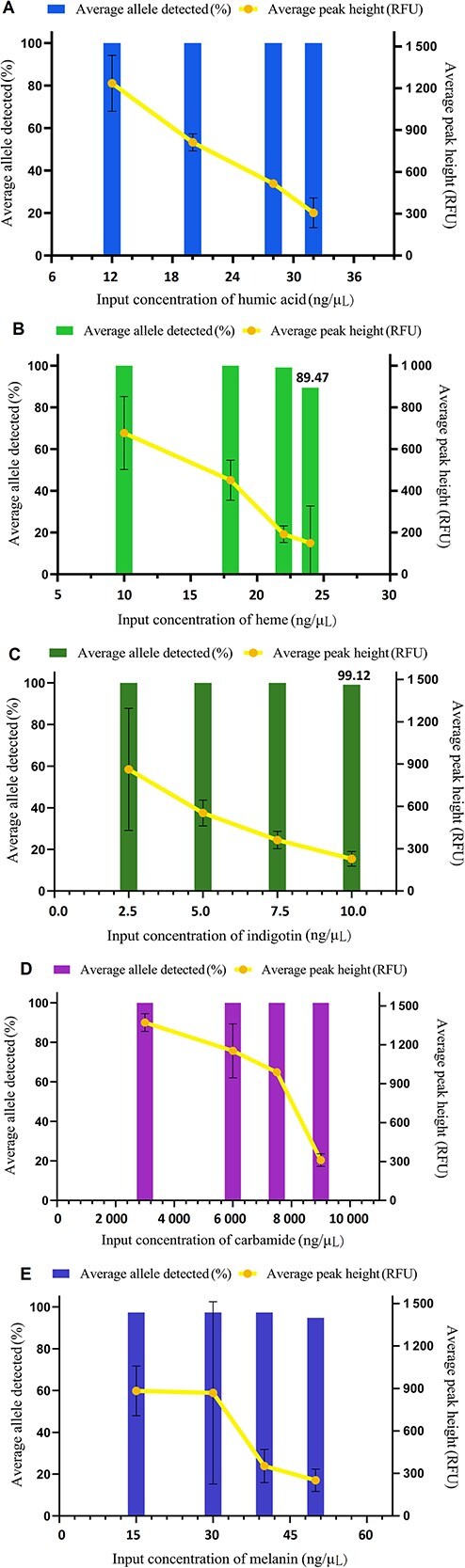
In the inhibitor study, 1 ng of control DNA 9948 was co-amplified with a series of concentrations of (A) humic acid, (B) heme, (C) indigotin, (D) carbamide, and (E) melanin, using the SureID^®^ X37 kit.

In forensic cases, samples such as bloodstains, body fluids, and hair are often collected from various environments and may contain inhibitors that can interfere with PCR reactions. We evaluated the stability of the 36 X-STR system using common PCR inhibitors. Overall, as the concentration of inhibitors added to the system increased, the number of detected alleles and the average peak height decreased. When the concentrations of humic acid, heme, indigotin, and carbamide were at or below 32, 22, 7.5, and 9 000 ng/μL, respectively, complete profiles were obtained from 1 ng of DNA 9948. However, when the concentrations of heme and indigotin increased to 24 and 10 ng/μL, respectively, the detected allele frequencies decreased to 89.47% and 99.12% (Figure 6A–D). Compared to previous studies [42], the SureID^®^ X37 system exhibited greater tolerance to these inhibitors. Within the detected concentration range of melanin, the average peak height ranged from 252.41 RFU (50 ng/μL) to 883.44 RFU (15 ng/μL) (Figure 6E). However, allele peaks with fragment lengths between 100 and 109 bp were replaced by non-target amplification fragments named “OL” (Supplementary Figure S9), which have also been reported in other literature [43], possibly due to the effect of melanin on metal ions and small molecules during the PCR process. These findings indicate that the new typing system can withstand a certain concentration of inhibitors.

### Heterozygote balance study

In heterozygous STR loci, two alleles have different amplicon sizes. Comparing the peak heights of these two alleles, known as “sister alleles”, can assist in interpreting mixed samples [44]. We observed that the PHR varied across different loci and allele fragment lengths. As shown in the Table 1, the mean PHR was 0.87107. The lowest PHR, 0.8060, was observed at the GATA172D05 locus, while the highest, 0.9196, was observed at the DXS10164 locus. Thus, this new genotyping system exhibited good balance across all loci, enabling accurate genotype interpretation.

### Stutter analysis

Stutter peaks are inherent outcomes of replication slippage during PCR and typically exhibit lower peak heights and fragment sizes similar to the target alleles (*n* ± 1 repeat units). When analysing mixed DNA profiles, these stutter peaks can be mistaken for alleles contributed by minor contributors, potentially complicating profile interpretation. Therefore, it is crucial to establish a stutter filter value for each locus [45, 46]. We conducted tests on 104 unrelated samples using this system to determine the stutter ratio, taking into account all possible stutter positions (*n* − 2, *n* − 1, *n* + 1). The results showed that stutters were predominantly absent at the “*n* − 2” and “*n* + 1” positions across most loci (Table 2). The highest stutter ratios at these two positions were both observed at DXS8377, with values of 0.0691 and 0.0622, respectively. At the “*n* − 1” position, all loci displayed stutter peaks, with the highest stutter ratio found at DXS8377 (0.2786) and the lowest at DXS10164 (0.0348). Moreover, except for DXS7424 and DXS8377, the mean stutter ratio at the “*n* − 1” position for all other loci was below 15%. Therefore, manual adjustments to genotypes may be necessary for these two loci as needed. In general, the average stutter ratios for all 19 coincident loci were lower compared to previous systems [40], facilitating the interpretation of mixture results.

**Table 1:** Peak height ratio was completed for 36 X-STR loci.

Marker	Counts	Mean	SD	Min	Max	PHR < 70%
DXS6795	78	0.8747	0.0848	0.7169	0.9941	0.00%
DXS7132	79	0.8613	0.0848	0.5994	0.9916	1.27%
DXS8378	57	0.8732	0.0847	0.7281	1.0000	0.00%
DXS10101	96	0.8732	0.0846	0.7191	0.9994	0.00%
DXS10103	84	0.8733	0.0846	0.5021	0.9993	8.33%
DXS10079	91	0.8862	0.0841	0.6504	0.9975	10.99%
DXS10134	87	0.8861	0.0843	0.6900	0.9933	1.15%
GATA165B12	54	0.8942	0.0838	0.7098	0.9996	0.00%
GATA172D05	78	0.8060	0.0838	0.6967	0.9996	1.28%
HPRTB	74	0.8696	0.0837	0.6764	0.9950	1.35%
DXS6810	55	0.8613	0.0791	0.7110	0.9943	0.00%
DXS10135	99	0.8629	0.0837	0.6254	0.9996	4.04%
DXS6797	73	0.8338	0.0831	0.6166	0.9970	6.85%
DXS10074	79	0.8836	0.0835	0.6219	0.9953	1.27%
DXS7424	69	0.8753	0.0833	0.7028	0.9981	0.00%
DXS9902	77	0.8829	0.0833	0.6522	0.9998	1.30%
DXS7423	56	0.8683	0.0832	0.3864	0.9981	1.79%
DXS10148	97	0.8164	0.0827	0.5227	0.9980	14.43%
DXS10162	78	0.8791	0.0815	0.6564	0.9978	1.28%
DXS6809	83	0.8661	0.0824	0.6850	0.9986	2.41%
DXS10159	79	0.8888	0.0824	0.6516	0.9986	1.27%
GATA31E08	74	0.8889	0.0823	0.6490	0.9984	1.35%
DXS6803	84	0.9059	0.0786	0.6629	0.9974	3.57%
DXS10075	77	0.8959	0.0822	0.7100	0.9969	0.00%
DXS6807	69	0.8433	0.0822	0.6458	0.9865	2.90%
DXS10164	64	0.9196	0.0816	0.7563	0.9988	0.00%
DXS6789	86	0.8578	0.0789	0.5207	0.9916	3.49%
DXS10146	91	0.8579	0.0821	0.5132	0.9986	8.79%
DXS7133	41	0.9044	0.0774	0.6560	0.9954	2.44%
DXS6804	81	0.8805	0.0818	0.6473	0.9973	2.47%
DXS981	75	0.8945	0.0807	0.7180	0.9996	0.00%
DXS9895	74	0.8570	0.0817	0.4334	0.9982	6.76%
DXS101	85	0.8704	0.0813	0.6585	0.9987	2.35%
DXS6800	31	0.8341	0.0676	0.6186	0.9838	6.45%
DXS9907	59	0.9006	0.0780	0.7655	0.9953	0.0000
DXS8377	97	0.8316	0.0812	0.3225	0.9983	0.0825

SD, standard deviation; PHR, peak height ratio.

**Table 2:** Stutter values were evaluated based on genotyping data from 104 unrelated individuals.

	*n* − 2 repeat units						*n* − 1 repeat units						*n* + 1 repeat units					
Marker	Counts	Min	Max	Mean	SD	Stutter filter	Counts	Min	Max	Mean	SD	Stutter filter	Counts	Min	Max	Mean	SD	Stutter filter
DXS6795	8	0.0075	0.0350	0.0209	0.0094	0.0491	143	0.0134	0.2458	0.0808	0.0382	0.1955	72	0.0096	0.0583	0.0376	0.0089	0.0643
DXS7132	22	0.0090	0.1034	0.0267	0.0189	0.0835	140	0.0274	0.4007	0.1052	0.0316	0.1999	16	0.0081	0.0560	0.0264	0.0170	0.0773
DXS8378	4	0.0087	0.0225	0.0151	0.0057	0.0321	110	0.0376	0.0936	0.0543	0.0126	0.0921	18	0.0081	0.1104	0.0207	0.0232	0.0903
DXS10101	–	–	–	–	–	–	121	0.0383	0.1073	0.0632	0.0120	0.0992	–	–	–	–	–	–
DXS10103	3	0.0079	0.0142	0.0115	0.0033	0.0212	138	0.0236	0.1141	0.0625	0.0174	0.1148	–	–	–	–	–	–
DXS10079	–	–	–	–	–	–	150	0.0434	0.1990	0.0804	0.0187	0.1364	2	0.0069	0.0979	0.0524	0.0643	0.2454
DXS10134	–	–	–	–	–	–	132	0.0331	0.1208	0.0674	0.0158	0.1147	–	–	–	–	–	–
GATA165B12	–	–	–	–	–	–	98	0.0261	0.1794	0.0516	0.0237	0.1227	11	0.0169	0.1369	0.0367	0.0343	0.1396
GATA172D05	4	0.0160	0.0339	0.0250	0.0082	0.0496	103	0.0293	0.1033	0.0600	0.0162	0.1085	22	0.0140	0.1032	0.0522	0.0283	0.1370
HPRTB	–	–	–	–	–	–	136	0.0206	0.1773	0.0984	0.0210	0.1614	–	–	–	–	–	–
DXS6810	–	–	–	–	–	–	107	0.0656	0.1662	0.0863	0.0124	0.1237	–	–	–	–	–	–
DXS10135	6	0.0112	0.0421	0.0287	0.0120	0.0647	175	0.0273	0.2336	0.0982	0.0244	0.1713	–	–	–	–	–	–
DXS6797	–	–	–	–	–	–	108	0.0501	0.2516	0.1344	0.0354	0.2405	–	–	–	–	–	–
DXS10074	–	–	–	–	–	–	82	0.0219	0.0939	0.0464	0.0124	0.0837	–	–	–	–	–	–
DXS7424	12	0.0263	0.0918	0.0566	0.0237	0.1276	128	0.0863	0.2672	0.1523	0.0128	0.1907	73	0.0348	0.2152	0.0606	0.0260	0.1387
DXS9902	–	–	–	–	–	–	119	0.0342	0.1737	0.0598	0.0155	0.1063	–	–	–	–	–	–
DXS7423	–	–	–	–	–	–	97	0.0279	0.0773	0.0528	0.0087	0.0791	–	–	–	–	–	–
DXS10148	5	0.0135	0.0691	0.0332	0.0216	0.0979	163	0.0237	0.1491	0.0800	0.0258	0.1574	–	–	–	–	–	–
DXS10162	–	–	–	–	–	–	118	0.0405	0.1567	0.0691	0.0177	0.1222	–	–	–	–	–	–
DXS6809	8	0.0207	0.0766	0.0538	0.0183	0.1086	148	0.0585	0.2751	0.1217	0.0394	0.2400	11	0.0163	0.0637	0.0401	0.0160	0.0881
DXS10159	–	–	–	–	–	–	60	0.0154	0.1295	0.0363	0.0170	0.0873	–	–	–	–	–	–
GATA31E08	4	0.0186	0.0509	0.0355	0.0155	0.0820	133	0.0289	0.2303	0.0657	0.0238	0.1370	6	0.0118	0.0742	0.0443	0.0232	0.1140
DXS6803	8	0.0100	0.1066	0.0361	0.0331	0.1354	141	0.0427	0.0915	0.0647	0.0091	0.0920	–	–	–	–	–	–
DXS10075	2	0.0389	0.0560	0.0474	0.0121	0.0836	118	0.0275	0.1712	0.0655	0.0183	0.1205	2	0.0113	0.0504	0.0309	0.0276	0.1137
DXS6807	–	–	–	–	–	–	91	0.0102	0.1128	0.0594	0.0157	0.1064	–	–	–	–	–	–
DXS10164	–	–	–	–	–	–	93	0.0197	0.0872	0.0348	0.0109	0.0676	4	0.0108	0.0272	0.0201	0.0079	0.0438
DXS6789	–	–	–	–	–	–	160	0.0531	0.1443	0.0933	0.0220	0.1592	–	–	–	–	–	–
DXS10146	–	–	–	–	–	–	143	0.0288	0.1011	0.0615	0.0161	0.1097	–	–	–	–	–	–
DXS7133	–	–	–	–	–	–	98	0.0261	0.0588	0.0381	0.0058	0.0555	–	–	–	–	–	–
DXS6804	–	–	–	–	–	–	141	0.0114	0.1239	0.0678	0.0201	0.1280	11	0.0184	0.0471	0.0295	0.0111	0.0629
DXS981	11	0.0089	0.0887	0.0469	0.0315	0.1414	133	0.0494	0.1768	0.0835	0.0193	0.1414	39	0.0120	0.1005	0.0402	0.0217	0.1052
DXS9895	8	0.0112	0.0524	0.0360	0.0144	0.0793	122	0.0403	0.1314	0.0689	0.0171	0.1202	14	0.0105	0.0724	0.0219	0.0167	0.0719
DXS101	28	0.0141	0.1330	0.0482	0.0312	0.1418	156	0.0448	0.2737	0.1144	0.0336	0.2152	44	0.0189	0.1713	0.0505	0.0250	0.1255
DXS6800	–	–	–	–	–	–	28	0.0094	0.0834	0.0425	0.0251	0.1178	26	0.0070	0.0744	0.0375	0.0192	0.0950
DXS9907	–	–	–	–	–	–	104	0.0304	0.0987	0.0476	0.0117	0.0828	–	–	–	–	–	–
DXS8377	37	0.0389	0.1415	0.0691	0.0199	0.1288	183	0.1818	0.3722	0.2786	0.0401	0.3989	3	0.0595	0.0644	0.0622	0.0025	0.0698

Bloded content indicates that the average stutter ratio is >15%, while “–” indicates that no stutter peak was observed at this locus.

### Population study

In this study, we successfully obtained the genotypes of 36 X-STR loci in a cohort of 577 Chinese Han individuals, comprising 316 males and 261 females (Supplementary Table S2). The genotyping was conducted in accordance with the “Specification of X-STR Testing for Forensic Purpose (SF/ZJD0105006-2018)” issued by the Bureau of Public Legal Services Administration, Ministry of Justice of the People’s Republic of China. After Bonferroni correction, we did not observe any significant deviation from HWE in the 261 female samples (*P* > 0.05/36).

Among the 630 paired loci analysed, 181 pairs showed significant deviations from LD in males after applying the Bonferroni correction (*P* < 0.05/630) (Supplementary Table S3). Notably, the DXS10101–DXS10103 pair also exhibited linkage in females (Supplementary Table S4). Some of these locus pairs displaying LD have been observed in previous studies as well [10, 40, 41, 47–49]. The formation of genetic linkage between loci is influenced by a complex interplay of factors, including physical/genetic distance and population genetic structure shaped by population mixing/stratification, making the establishment of LD a multifaceted process [50, 51]. Consequently, further pedigree studies are warranted to investigate the linkage between markers and tightly linked haplotypes. Based on the statistical significance of the DXS10101–DXS10103 pair in both male and female LD tests in this study, we computed the corresponding genetic parameters based on haplotype frequencies rather than allele frequencies [52], and the results revealed a total of 67 observed haplotypes for DXS10101–DXS10103 (Supplementary Table S5), with frequencies ranging from 0.0032 to 0.0949. Both the PIC and PD values exceeded 0.96 (Supplementary Table S6), highlighting the high polymorphism and information content of DXS10101–DXS10103 in the Chinese Han population.

Herein, we detected a total of 347 alleles in females, with corresponding frequencies ranging from 0.0019 to 0.8506. Males displayed 324 alleles, with frequencies ranging from 0.0016 to 0.8133. Chi-square tests revealed no significant differences in allele frequency distribution between males and females (*P* > 0.05). Consequently, we calculated allele frequencies (Supplementary Table S7) based on the pooled population. Overall, we identified a total of 375 alleles, with frequencies ranging from 0.0009 to 0.8302. The number of alleles per locus varied, ranging from 5 (DXS7423) to 25 (DXS10135).

The forensic parameters of the 34 X-STR loci are presented in Supplementary Table S8. The average PIC value was 0.6940, with 31 loci demonstrating high levels of polymorphism (PIC > 0.5). Among these loci, DXS6800 displayed the lowest PIC value (0.2869), while DXS10135 exhibited the highest (0.9183), consistent with previous studies [11, 12, 39, 42, 47, 53, 54]. Notably, DXS6800 had the lowest PDm (0.3009) and PDf (0.4973), while DXS10135 boasted the highest PDm (0.9235) and PDf (0.9890). The combined PD and cumulative probability of exclusion for this system exceeded 0.999 999 999 999 997 (Supplementary Table S9). Collectively, this comprehensive set of markers demonstrated substantial forensic efficiency, underscoring the potential of this system as a robust supplementary tool for personal identification and kinship determination in the Chinese Han population.

## Conclusion

In this study, we validated the 36 X-STR multiplex system following both SWGDAM guidelines and the Chines national standard GB/T 37226-2018 and confirmed the kit’s accuracy, reliability, and sensitivity in the precise detection and identification of mixed samples, underscoring its promising applicability in forensic. Population investigations confirmed its high efficiency in the Chinese Han population, making it a potent tool for personal identification and kinship determination. Nevertheless, we acknowledge the need for future pedigree investigations to comprehensively assess LD and recombination rates among these X chromosomal markers for ongoing refinements in forensic applications.

## Supplementary Material

Figure_S1_owae029

Figure_S2_owae029

Figure_S3_owae029

Figure_S4_owae029

Figure_S5_owae029

Figure_S6_owae029

Figure_S7_owae029

Figure_S8_owae029

Figure_S9_owae029

Supplementary_Table_legend_owae029

Supplementary_Table_owae029
