# Supplementary figures and images for: Validation and forensic application of a new 36 X-chromosomal short tandem repeat loci multiplex system
Source: Forensic Sci Res. 2024 Apr 23;10(2):owae029. doi: 10.1093/fsr/owae029 (PMC12140019; doi:10.1093/fsr/owae029)

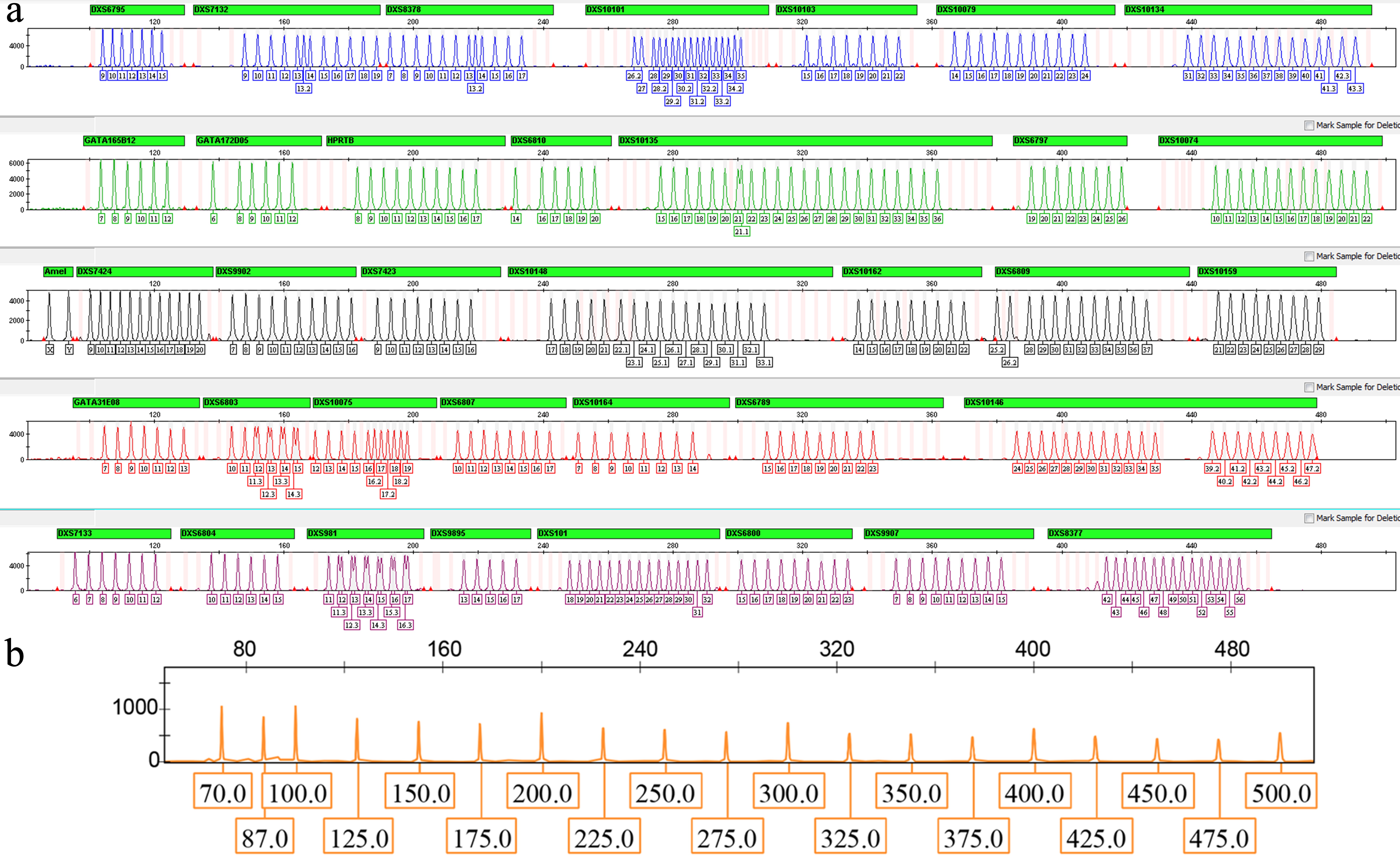

Supplement: Figure_S1_owae029 [file figure_s1_owae029.jpeg]

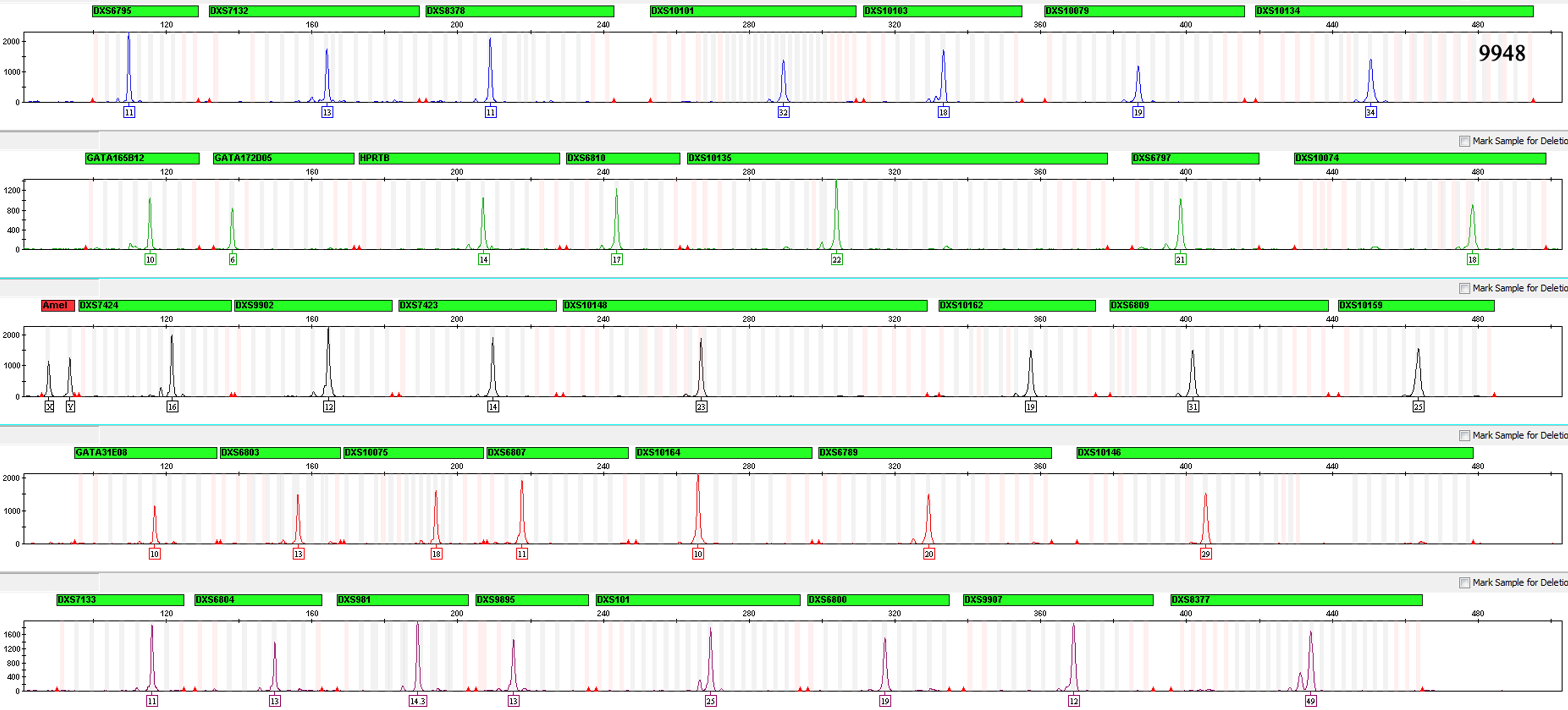

Supplement: Figure_S2_owae029 [file figure_s2_owae029.jpeg]

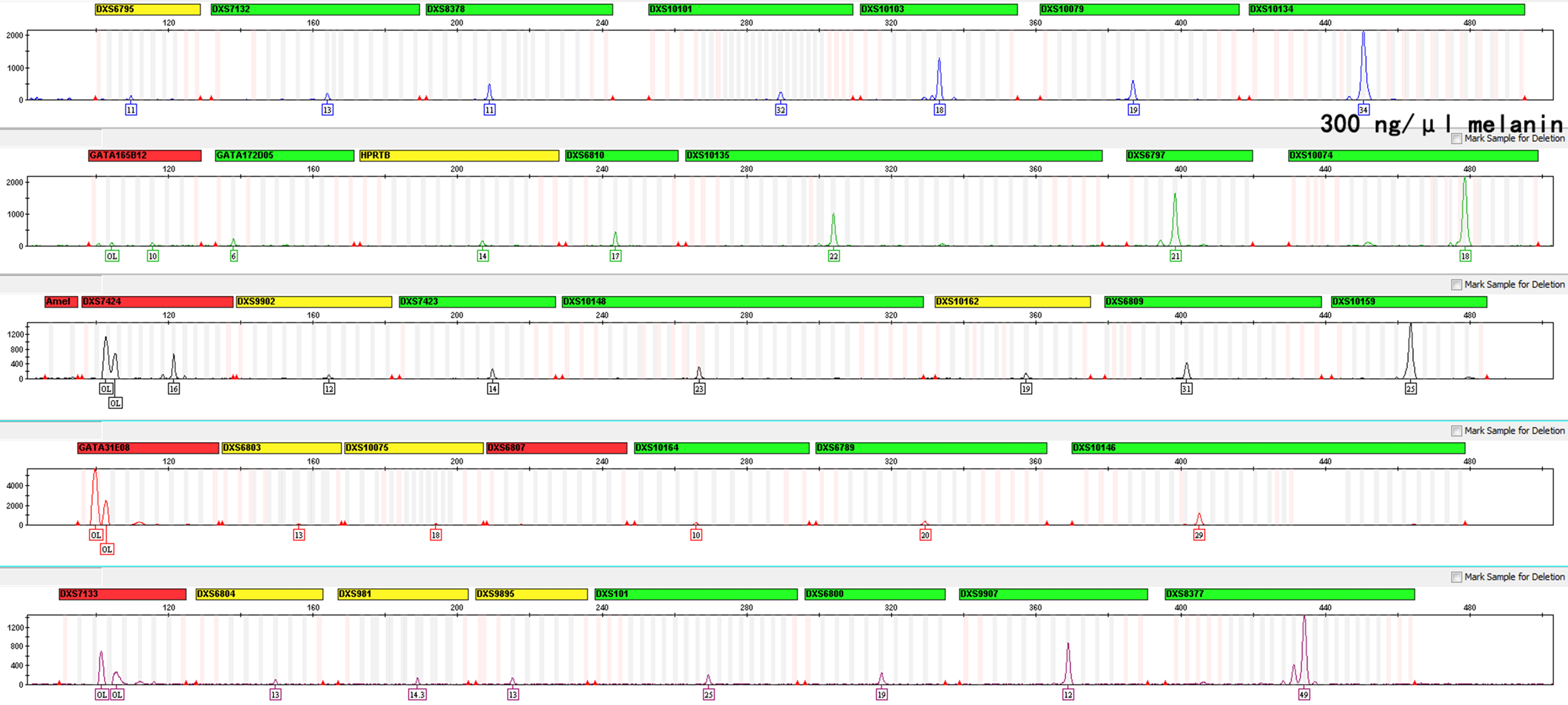

Supplement: Figure_S9_owae029 [file figure_s9_owae029.jpeg]
