# Supplementary material for: Validation and forensic application of a new 36 X-chromosomal short tandem repeat loci multiplex system
Source: Forensic Sci Res. 2024 Apr 23;10(2):owae029. doi: 10.1093/fsr/owae029 (PMC12140019; doi:10.1093/fsr/owae029)
Supplement: Supplementary_Table_legend_owae029 [file supplementary_table_legend_owae029.docx]

**Supplementary Figure legends**

Supplementary Figure S1. Electropherograms of Allelic ladder (A) and internal standard (B) of the SureID^®^ X37 Kit.

Supplementary Figure S2. Electropherogram of control DNA 9948 of the SureID^®^ X37 Kit.

Supplementary Figure S3. Electropherograms of different annealing temperatures for SureID^®^ X37 kit. From A to E were 59℃, 60℃, 61℃, 62℃ and 63℃, respectively.

Supplementary Figure S4. Electropherograms of different cycles of PCR for SureID^®^ X37 kit. From A to E were 24, 26, 27, 28, 30 cycles, respectively.

Supplementary Figure S5. Electropherograms at different terminal extension time for SureID^®^ X37 kit. From A to D were 0min, 15min, 30min,45min, respectively. The alleles with a red box in the figure indicate the presence of an “- A” peak.

Supplement Figure S6. Electropherograms of three different aged samples of the SureID^®^ X37 Kit. From A to C, there were blood spot samples saved on blood cards for 1 year, over 6 years, and on tissue paper for over 13 years, respectively.

Supplementary Figure S7. From A to D, there were electropherograms of the mixtures of DNA 9948 and 9947A at the ratio of 4:1, 2:1, 1:1, 1:2 and 1:4 of SureID^®^ X37 Kit, respectively.

Supplementary Figure S8. Electrophergrams of non-huaman samples of the SureID^®^ X37 Kit. From A to K, there were cat, chicken, horse, cow, rat, rabbit, sheep, pig, dog, goose and macaque, respectively.

Supplementary Figure S9. Electrophergram of 300 ng/μL melanin of the SureID^®^ X37 Kit.

**Supplementary Table legends**

Supplementary Table S1. The information of SureID^®^ X37.

Supplementary Table S2. Genotypes of the 37 STR loci of 577 unrelated Han individuals typed with the SureID^®^ X37 typing system (261 females, 316 males).

Supplementary Table S3. The *P*-values of LD testing for 36 X-STR loci of the Chinese Han population (316 males).

Supplementary Table S4. The *P*-values of LD testing for 36 X-STR loci of the Chinese Han population (261 females).

Supplementary Table S5. The haplotype and corresponding frequencies of DXS10101-DXS10103 in Chinese Han population.

Supplementary Table S6.The forensic population genetic parameters of DXS10101-DXS10103.

Supplementary Table S7. Allele frequencies of 34 X-chromosomal STR loci in 577 individuals of the Chinese Han population.

Supplementary Table S8. The forensic population genetic parameters of 34 X-STR loci based on 577 unrelated individuals.

Supplementary Table S9. The forensic efficiency parameters.
